# Social rank and social cooperation: Impact of social comparison processes on cooperative decision-making

**DOI:** 10.1371/journal.pone.0175472

**Published:** 2017-04-07

**Authors:** Xu Gong, Alan G. Sanfey

**Affiliations:** 1 Donders Institute for Brain, Cognition and Behaviour, Radboud University Nijmegen, Nijmegen, The Netherlands; 2 Behavioural Science Institute, Radboud Univeristy Nijmegen, Nijmegen, The Netherlands; Tianjin University of Technology, CHINA

## Abstract

Successful navigation of our complex social world requires the capability to recognize and judge the relative status of others. Hence, social comparison processes are of great importance in our interactions, informing us of our relative standing and in turn potentially motivating our behavior. However, so far few studies have examined in detail how social comparison can influence interpersonal decision-making. One aspect of social decision-making that is of particular importance is cooperative behavior, and identifying means of maintaining and promoting cooperation in the provision of public goods is of vital interest to society. Here, we manipulated social comparison by grading performance rankings on a reaction time task, and then measured cooperative decisions via a modified Public Goods Game (PGG). Findings revealed that individuals ranked highest tended to be more cooperative as compared to those who placed in the bottom rank. Interestingly, this effect was regardless of whether the comparison group members were the subsequent players in the PGG or not, and this effect was stronger in those with higher social orientation. In summary, the present research shows how different social comparison processes (assessed via social rankings) can operate in our daily interaction with others, demonstrating an important effect on cooperative behavior.

## Introduction

Social comparison is an extremely important factor in how we interact with others. This process provides a direct evaluation of where we stand in relation to others, and provides an indication of the characteristics of ourselves that we should modify in order to improve in a variety of ways. In the animal world, social rankings and hierarchies are ubiquitous, and primates, in particular, are quite adept at comparing themselves to one another via social rankings, a capacity that has important consequences for successful group living [1]. The human world is no less sensitive to hierarchies. There is a strong tendency to compare oneself with others, to estimate and tag others, and then categorize them along a spectrum of importance, often termed “social status,” in view of how valuable we perceive that person to be. Those whom we deem as high value like politicians, celebrities, athletes, film stars, and so forth, we place at the top of our social rankings, while those we typically consider as low value fall to the bottom of our social hierarchy [2]. Successful navigation of our complex social world demands an ability to identify and estimate the relative status of others, and knowledge about others’ standings can help us to optimize our own social value.

A considerable amount of previous experimental work has demonstrated a clear and strong relationship between social comparison and behavior. For example, players who exhibited relatively better performance during social interactions were judged by others as worthy of a larger prize [3]. We can, therefore, talk about ‘downward’ social comparison, when we are better off than others, as well as ‘upward’ social comparison when we are worse off than our peers. Social comparison seems, therefore, natural, but when coupled with behavioral or affective consequences it can have striking effects on decision-making [4–6]. For example, households perceived to be economically worse off in comparison to others reported less purchasing decisions for durable goods, and thought more seriously about purchasing decisions [7]. In a lottery task, individuals who experienced a large social gain in early trials as compared to others subsequently increased their risk-seeking behavior in the following trials [8, 9]. In short, social comparison processes illuminated the interpersonal concern by overshadowing the concern for personal outcomes independent of others. Given the impact of social comparison on even quite fundamental decisions outlined above, such as consumer purchases or risk choices under uncertainty, we might expect that decisions made in an interactive social context [10] would be even more susceptible to the influence of our social comparative processes, and indeed social information has been shown to influence decisions in a wide variety of domains [11]. Comparison of recycling behaviors between oneself and the ‘average’ person altered households’ subsequent recycling decisions [12], as well as towel (re)use in hotels [13]. Individual perception of the seriousness of one’s own legal infringement in terms of downloading software from the internet depends on beliefs about one’s position within the distribution of illegal downloaders in the UK, rather than the objective positions[14]. This evidence suggests that that social comparison holds considerable sway in social decision-making in our daily life.

An important social decision that we are often faced with is whether or not to cooperate with others. Successful cooperation offers considerable benefits for ourselves and for others, but sometimes places us at risk of our cooperative acts being abused, and can be therefore a risky choice option. Ensuring sustained cooperation is challenging because cooperation sometimes has a price, namely that one can be taken advantage of by a so-called ‘free-rider’. Despite this, human cooperation plays a vitally important role in the development and functioning of society [15]. A unique distinction of the human species is that they shape social life by minimizing selfish behaviors and developing cooperative agreements with normative responsibilities and obligations [16, 17]. Nevertheless, the origin of cooperation, especially in a competitive world, is still somewhat of a puzzle for philosophers, economists, psychologists, and neuroscientists [18–21]. Hence, exploring and recognizing individual and situational factors that help in understanding cooperative behaviors is of great importance.

The Public Goods Game is an effective and useful tool to study mutual cooperation in the laboratory. [22]. This game models the willingness of players to contribute to the maintenance of a so-called ‘public good’, that is, a common resource that can freely be used by anyone without regard for whether one has contributed or not, for example, a public park. While the collective is best served by the existence of such a public good, each individual’s optimal choice is to withhold contribution and instead free-ride on the participation of others. This tension between public and private benefit is one extremely important consequence of cooperation. Ideally, everyone would gain a significant benefit based on mutual cooperation by contributing the maximum amount, but a self-interested tactic is to contribute nothing and reap the benefits of other’s cooperative acts. The PGG is an effective and useful tool to study mutual cooperation, and research has found that incentives such as reward and punishment are successful at promoting cooperative behavior within the group [23, 24]. Nonetheless, despite extensive use of this experimental task, there have been few explicit tests of how social comparison can impact cooperative behavior in this particular context.

Therefore, the purpose of this study is to explore how social comparison can affect cooperative decision-making, and to gain an insight into psychological processes underlying these effects. In order to achieve this goal, we address three specific research questions. Firstly, and most importantly, if social comparison indeed describes how we stand in relation to others, as we have outlined above, then how might different types of social comparison (i.e. upward or downward) affect and modify interpersonal decisions in a cooperative context? As described previously, evidence from a variety of studies suggests that there are robust effects of social comparative processes on purchasing decisions, risk choices, and fairness perceptions. Taken together, relative performance can initiate a downward or upward comparative process, which we would then expect to impact subsequent cooperative decisions [25].

Secondly, people usually have beliefs about the typical behaviors of others in societies, namely social norms, and often adjust their own behavior in accordance with this information. Therefore, beliefs about specific others might also be relevant to decision-making, especially in a social context [26]. Assuming social comparison processes do affect cooperative decision-making, we were also interested in knowing how these regulatory behavioral effects were influenced by specific knowledge about those with whom one must cooperate. To answer this research question, we manipulated participants’ beliefs about their cooperative partners. In one case, they had knowledge about how the players in their cooperative group ranked relative to themselves—that is, the cooperative group were the same players as those they had previously competed with, and had been directly compared to (Relevant targets). In the second condition, the cooperative group was comprised of new players chosen after the social comparison task, that is, the participant had no direct knowledge of how he or she ranked relative to these players (Irrelevant targets). This allows us to better understand whether social comparison subsequently affects interactions only with those we know our rankings relative to, or whether the affective and cognitive processes altered by knowing one’s rank can have a more general impact on behavior, irrespective of who we interact with.

Finally, we are also interested in individual sensitivity to social comparison [27]. Social comparison orientation (SCO), as defined by Gibbons and Buunk [28], refers to the tendency to compare oneself to others [29]. Previous research found that high SCO individuals reported relatively more positive effect after downward comparison and more negative affect after upward comparisons when they perceived a cooperative social climate at work[30]. This evidence shows that individual’s social comparison orientation tendencies would give rise to more salient effects on the subsequent interpersonal outcomes. To this end, we will assess individual differences in social comparison orientation by using the Iowa-Netherlands Comparison Orientation Measure (INCOM) [28], and examine how this measure corresponds to cooperative decision-making under conditions of both upward and downward social comparisons.

We addressed these research questions by designing a paradigm in which participants would receive feedback about their performance in a task, in a social context, and then we subsequently observed their willingness to cooperate in an ostensibly separate task. To generate social comparison, participants completed a simple reaction time task at the same time as 4 other players. After a button press in response to a color-cue, participants were informed where they ranked amongst the 5 players, with the particular conditions of interest being rank #1, rank #3, and rank #5, corresponding to downward, neutral and upward comparison conditions, respectively. After this manipulation, participants then played a standard PGG with either the 4 players they had been ranked against, or with 4 new, unranked, players. This allowed for an examination of whether any comparison effects were limited to those players who were actually in the comparison group, or whether they extended to novel players. In addition, participants played a third set of PGGs without a prior ranking task in order to assess their baseline levels of cooperation.

## Methods

### Participants

Participants were 39 college students from Radboud University Nijmegen, with an age range from 20 to 30 years (M = 23.03, SD = 2.39). They were recruited via advertisements informing them that they would be playing a decision-making game, and were compensated 8 euro for participation. There was an additional 8 euro payment possible based on their performance. On average, participants earned 4 euro as a bonus payment. The study protocol was approved by the local ethics committee (CMO region Arnhem-Nijmegen, The Netherlands) under the general ethic approval (CMO 2014/288), and all the experimental methods were conducted in accordance with these guidelines. All participants provided written informed consent in accordance Declaration of Helsinki and the guidelines of local ethics committee.

### Procedure

#### Instructions and practice

Participants were instructed as to the nature of the experiment. They were told that over the course of the experiment they would be paired up with other participants who had previously taken part in the study. They were informed that their own responses would be used by later participants, but that this data would be completely anonymized. It was explained that on each trial of the experiment, participants would be paired with 4 other (anonymous) players, and that they would never play with the same set of players twice. They were told that they could receive a monetary bonus of (maximally) 8 euros in total; 6 euros based on performance in the Public Goods Game (PGG: see below), and 2 euros based on average ranking in the Circle task (see below). After that, they practiced the Circle task, and then saw six practice trials of the PGG, with all conditions practiced. They were allowed to ask questions during the instructions and practice session, and the experiment did not begin until it was clear that they understood the instructions.

#### Social comparison manipulation: Circle task

**Fig 1:**
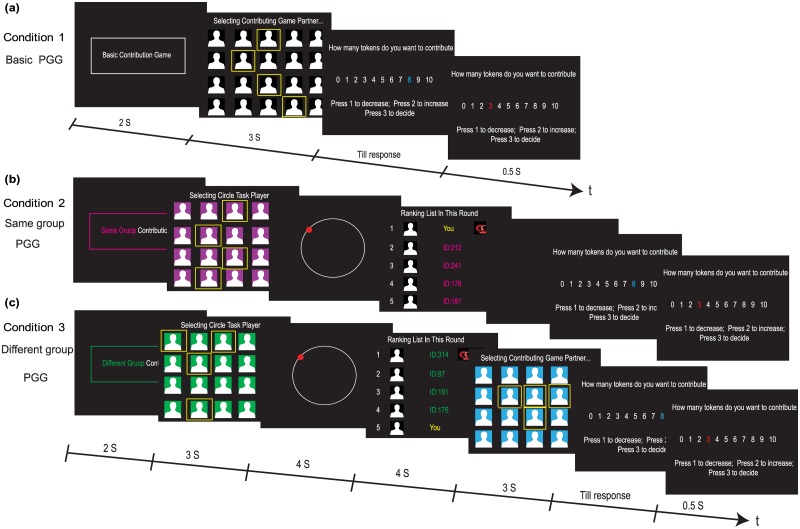
Experimental tasks and timelines. (**a**) A single trial of the basic public goods game. In this condition, participants are randomly paired with 4 anonymous partners to play the standard public goods game, (**b**) A single trial of the same group public goods game. In this condition, participants are randomly paired with 4 anonymous partners for the circle task, and then play the public goods game with the same set of people, (**c**) A single trial of the different group public goods game. In this condition, participants are first randomly paired with 4 anonymous partners to play circle task, and then paired with another 4 partners to play the public goods game.

A simple perceptual task was used to manipulate social comparison. In the circle task, a small colored circle moved around the periphery of a larger static white circle. In each trial, the start color of the small circle was to be randomly assigned from a color pool of red, purple, blue, green, pink and yellow. The color of the small circle was then randomly replaced by another color in the pool at a random interval of between 0.64–0.8 seconds. The task for the participant was to press any key on the keyboard as soon as they detected that the color of the small circle had changed. Participants were informed that their performance measure was based on both the accuracy and the speed of their response. Following each trial of the task, a ranking list was generated based on the performance of 5 purported players in the task. In reality, this ranking list was pre-programmed so that the participant appeared systematically at each ranking. Participants were ranked from #1 to #5, with higher likelihoods of ranks 1, 3, and 5. The rankings were determined randomly, with the proviso that rank 1 or 5 was automatically assigned if the response time was less than 0.05 s or larger than 1 s respectively (Fig 1).

#### Cooperation measure: Public goods game

A Public Goods Game was used to measure cooperation. In each trial of the game, participants were randomly paired with 4 other, anonymous, players. Participants were endowed with 10 tokens at the start of each trial and had to decide how many of these tokens they wanted to contribute to the group account and how many of the tokens they wanted to keep for themselves. Once all the players decided how much to contribute, the total contribution to the group account was then multiplied by 1.6, and this final amount was divided equally across all 5 group members. Earnings in each trial are therefore the sum of the tokens participants did not contribute to the group account (initial tokens minus contribution) plus the payment from the group account (aggregated contributions to group account multiplied by 1.6, then divided by 5).

#### Behavioral testing

Three conditions were measured in the experiment, each consisting of 12 trials, for a total of 36 trials (Fig 1). Condition 1 (Basic-PGG) provided a baseline, with participants playing the Public Goods Game with 4 other anonymous partners. In Condition 2 (Same Group-PGG) and Condition 3 (Different Group-PGG), participants first played the Circle Task with 4 other anonymous partners. Following each trial of the task, a ranking list was generated based on the performance of the 5 purported in the task. In reality, this ranking list was pre-programmed so that the participant appeared systematically at each ranking. Participants were ranked from #1 to #5, with higher likelihoods of ranks 1, 3, and 5. After participants have seen the ranking list, in Condition 2 (Same Group-PGG), participants immediately followed by playing the PGG with the same set of partners, whereas in condition 3 (Different Group-PGG), participants then played the PGG with a different set of 4 partners. In both Condition 2 and 3, on average, players were ranked #1 3 times, #3 3 times and #5 3 times. We randomized the entire 36 trials, consisting three experimental conditions, for each participant.

The first condition allows us to measure a baseline performance in a PGG, and then compare that performance to the other conditions where social ranking information is presented immediately before contribution decisions. The latter two conditions then enable us to distinguish between situations where the rankings are relevant for the cooperative decision (Same Group condition) or where they are irrelevant for that choice (Different Group condition).

#### Post experiment questionnaire

After completion of the experiment, participants filled out two short questionnaires. The first consisted of questions about the experimental procedure. The next was the Iowa-Netherlands Comparison Orientation Measure (INCOM) [28], which was used to examine individual differences in social comparison orientation. Afterwards, there was a short interview about the strategy used during the experiment. Finally, a selection of trials was randomly chosen for each participant and randomly paired with choices from four other real participants to calculate their Public Goods Game bonus. Participants received an average of 1 euro for their Circle Task bonus, as these rankings were largely predetermined.

## Results

### Individual’s baseline-level of cooperative behaviors

**Fig 2:**
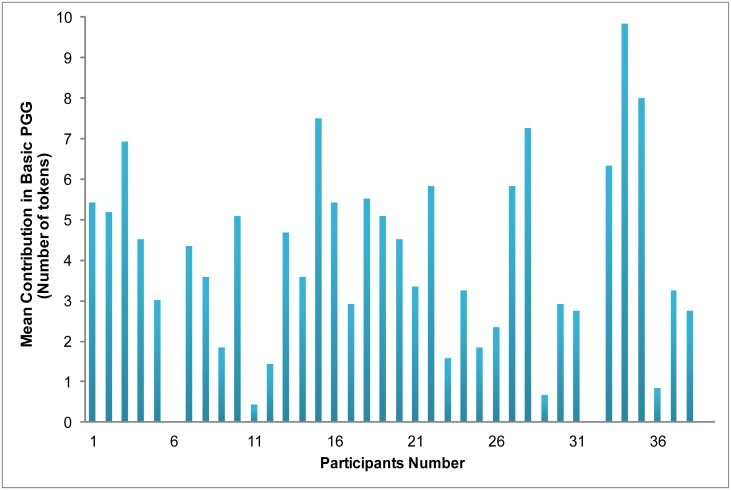
Individual differences of contribution in the baseline condition—Basic PGG.

We first examined performance in the Basic Public Goods Game (where no competitive rankings were supplied) as an indicator of the general type of cooperative behavior exhibited in the task. Descriptive statistics of the contribution amount in this condition demonstrated considerable individual differences in behavior, though almost all participants contributed something on average (*M* = 3.83, *SD* = 2.91, 3 always contributed zero, and 1 contributed the maximal amount in every round) (see Fig 2).

### Effects of experimental conditions on cooperative behaviors

**Fig 3:**
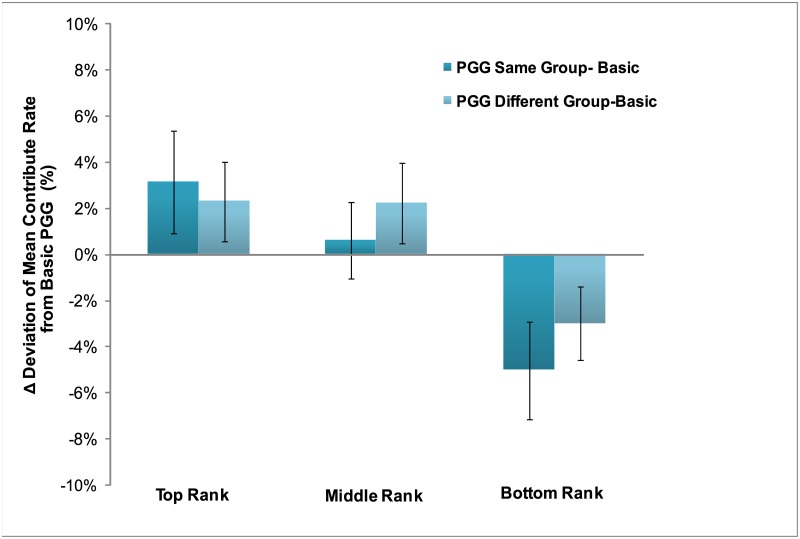
Deviation of mean contribution rate from the basic public goods game in the same and different group conditions across ranks.

The main purpose of this study is to examine how the rankings and groups influence cooperative decisions, as well as the role of social comparison orientation. Firstly, for analysis of the cooperative decisions, we used each participant’s contribution amount in the Basic Public Goods Game as a baseline measure, and then computed the deviations from that amount for each of the conditions of interest, namely the two other Public Goods Games (Same group and Different group) for each of the three ranking levels (#1, #3, #5). A 2 × 3 × 2 three-way mixed repeated analysis of variance (ANOVA) was implemented including group (same, different) and rank (top, middle, and bottom) as the within-subject factors and social comparison orientation tendency (High Social Comparison Orientation group, HSCO; and Low Social Comparison Orientation group, LSCO) as the between-subject factor. Fig 2 provides an overview of the results. The results show that the contribution amount was significantly affected by the rankings in both the Same and Different group conditions, *F* (2, 74) = 6.12, p<0.01. Further analyses across the different ranking conditions found that there was a significant linear trend, *F* (1, 37) = 8.43, p<0.01, indicating that as the rank increased, contribution in the both Same and Different group Public Goods Game increased proportionately, with the contribution when top-ranked (M = 2.73%, SD = 1.61%) greater than that when middle ranked (M = 1.42%, SD = 1.43%), which in turn was greater than that when the bottom ranked (M = -4.00%, SD = 1.59%). However, there was no main effect of group (F (1, 37) = 0.42, p = 0.52), and also no interaction was found (see Fig 3).

### Individual differences of social comparison orientation on cooperative behaviors

We are further interested in individual differences, and how sensitivity to social rank might play a role in cooperative decisions. For the Iowa-Netherlands Comparison Orientation questionnaire data analysis, the median of participants’ social orientation questionnaire score was 40 (M = 39.13, SD = 6.04), which was used as the threshold to separate the participants into LSCO (N = 20) and HSCO (N = 19). An independent-sample t-test showed that the social comparison score in the HSCO group (M = 43.95, SD = 2.68) was indeed significantly higher than the LSCO group (M = 34.55, SD = 4.56), t (37) = -7.80, P<0.001.

**Fig 4:**
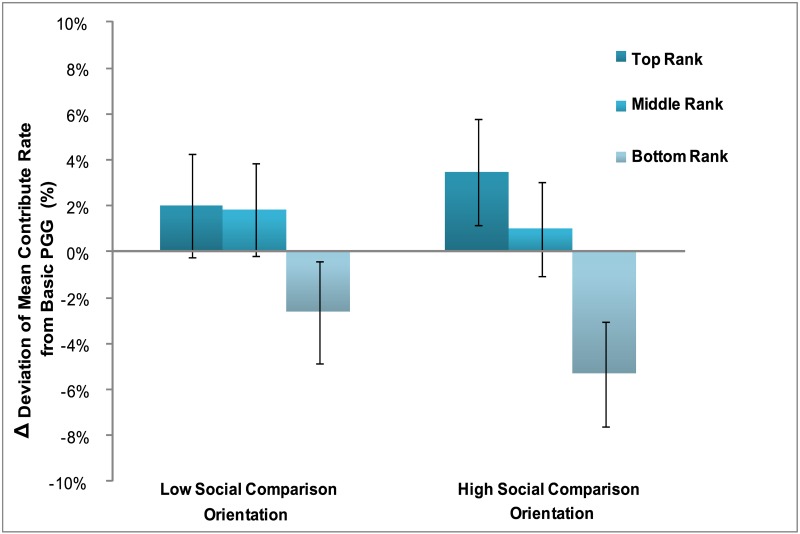
Deviation of mean contribution rate from basic public goods game in the same and different group across low social comparison orientation and high social comparison orientation group.

To test how manipulated rankings impacted individuals with different social comparison orientation. A 2×3 mixed repeated ANOVA was implemented, including social comparison orientation (LSCO, HSCO) as a between-subject factor, rank (top, middle, and bottom) as the within-subject factor, and the contribution deviated from baseline condition as independent variable. Greenhouse-Geisser correction was carried out as the spherical hypothesis was violated. We found that the main effect of rank was significant in the HSCO group, F (2, 36) = 8.97, p < .001, though not in the LSCO group, F (2, 38) = 1.20, p = .31 (see Fig 4).

## Discussion

In this study, we investigated the effects of social comparison on cooperative behavior. We hypothesized that social comparison, via its role in the assessment and evaluation of the self and the others, would affect social decision-making. The particular decisions we were interested in were choices about how much to cooperate with others, in particular when this cooperative choice comes with the risk of exploitation by free-riders. Firstly, we were interested in the respective effects of upward and downward social comparison on cooperative decisions. The former refers to a comparison with those who are relatively better off than we are, while the latter occurs when we compare ourselves to those worse off. Secondly, we also explored how these social comparison effects impacted both ‘relevant’ and ‘irrelevant’ targets. Here we defined relevant targets as partners in the cooperation task with whom participants had been previously directly compared with on simple cognitive task, whereas irrelevant targets were those partners in the Public Goods Game with whom there was no previous comparison provided. Finally, we examined how individual differences in social comparison orientation, as assessed by an independent measure, could potentially underlie these effects.

Our primary findings of interest were that, firstly, when people earned a place in the top rank of players, thus inducing downward social comparison, we observed a significant increase in cooperative behavior in the subsequent PGG, as compared to participants’ own baseline level of cooperation when playing without any social information. In contrast, when people were in the bottom rank, resulting in upward social comparison, their cooperative behavior decreased. Importantly, these effects did not depend on whether the comparisons were with relevant (Same group condition) or irrelevant targets (Different group condition), with similar results in evidence for each set of targets. Finally, when examining individual differences in these changes in cooperative behavior, we found these effects were strongest in participants with high social comparison orientation scores.

Regarding our first research finding, on trials in which individuals achieved a higher rank on the cognitive task, on average they made more cooperative decisions, that is, they contributed significantly more to the public good than on those trials where they were placed at lower ranks. Additionally, while we found that higher social ranks enhanced cooperative actions, as described above, conversely, our results demonstrated that finding oneself ranked bottom of the list led to a significant decrease in cooperation. As showed in our task, the Circle task (ranking process) and the Public Goods Game (decision process) are the separate and independent task. Therefore, rational individuals’ decisions in the public goods game according to to the classic economic models should not be influenced by the rankings in the previous circle task. These interesting effects can potentially be explained in several ways. Firstly, higher rankings imply an advantageous position and represent higher competence, which is likely experienced as a social reward in most societies [31–34]. This social reward process may well have a generally positive impact on mood (similar to ‘warm-glow’) [35, 36] which could in turn lead to alterations in subsequent behavior, such as higher contributions in the following PGG [35, 37, 38]. In a similar vein, the negative emotion experienced by low ranked status (e.g., frustration, sadness) could elicit less willingness to engage in social interactions [39, 40] and thus fewer cooperative decisions in the subsequent PGG. An alternative account is that relative performance derived from the social comparison task might lead to an advantageous or disadvantageous social experience during the interactions with other players, which could then enter into the subsequent decision-making via social or interpersonal utility [11]. According to the inequality aversion model [20], individuals have a robust aversion to disadvantageous inequality in situations where they are confronted with poorer performance or outcomes in comparison to others. In addition, many people also demonstrate an aversion to advantageous inequality, that is, avoiding outcomes where they are made considerably better off than their game partner. This inequality aversion explanation suggests that fairness considerations about the relative performance of players on the cognitive task may come into play [41, 42]. Thus, individuals’ social preferences of inequality aversion might modify the subsequent cooperative decisions in an interpersonal context [20, 42]. Specifically, a strong distaste for disadvantageous inequality might decrease their interpersonal utility, which would explain why individuals at lower ranks decrease their subsequent cooperative behaviors in the PGG; whereas individuals’ reluctance to assume the position of advantageous inequality might increase their social utility, which can explain why individuals at higher ranks raise their contribution in the succeeding PGG. A third plausible explanation for our findings is that the increased cooperative behaviors observed by the higher ranked players might be motivated by considerations of avoiding anticipated negative emotions caused by (e.g., guilt)[43]. By this account, individuals with a higher rank might feel that cooperative behavior is expected of someone who is a ‘winner’, and this social norm may guide subsequent decisions to be more generous, and thus avoid the guilt associated with taking further advantage of one’s game partners[26]. Conversely, task ‘losers’ may subsequently decide to maximize their own profit in the game, and thus, contribute relatively less.

Given that there was a Circle task bonus, which participants were informed they could earn additional 2 euros if their average ranking across all the social comparison trails were above rank 3, one may argue that a simple non-social motivation to maximize financial outcome could have played a role in the changes from the baseline in the average contribution in the other two social comparison PGG tasks. In our opinion, firstly, the 2 euros bonus was based on the average ranking in the Circle task across all the social comparison trails that participants would not experience any monetary gain experience in a single trail during the task. Therefore, the social comparison effect should play a dominant role in the modification the motives for the following contribution tasks. More importantly, if this argument is true, which means potential monetary reward would occur in rank 1 and 3 but not in rank 5 in a single social comparison trial, we should observe the effect in condition rank 1 and 3 rather than rank 5 (supposed to be no effect at all). However, in fact, our results showed that the experience of bottom rank (rank 5) during the Circle task decreased their contribution in subsequent PGG, which demonstrated the social comparison effect.

A second important result which can help shed light on the potential mechanisms of cooperation outlined above emerges from the two separate ranking conditions we employed here. In one condition, participants took part in the PGG with players they had previously been directly ranked against (‘relevant’ targets), while in a second condition they played the PGG with a different group than those they had been ranked with (‘irrelevant’ targets). Nonetheless, participants showed very similar patterns of cooperation across both conditions (greater cooperation for downward comparison, lesser cooperation for upward comparison). Given what we have discussed above about the inequality aversion model, one might hypothesize that the social comparison processes would have a greater impact on individuals’ contribution in PGG with relevant as opposed to irrelevant targets. However, participants tended to treat the two groups the same way regardless of ‘relevant’ or ‘irrelevant’ compared targets. Further, if players’ beliefs about the expectation of others would lead to changes in subsequent cooperative decisions, then one might again expect that this would occur to a larger extent in those they had been ranked against, as opposed to a set of players with whom they had no previous experience. Therefore, we believe this most plausible mechanism for the alterations in cooperative decisions as a function of social comparison is one of affective bias. The positive emotions from a ‘winning’ rank enhance cooperation independent of whomever one is engaged in the PGG with, while a ‘losing’ rank leads to indiscriminately lowered cooperative choices. These results can be integrated with findings of pro-social (e.g., charity donation) decisions, in which research found the neurobiological evidence of reward processing when people prosocially interact with others [44–46]. Moreover, this was supported by studies of social exclusion, whereby people in a disadvantageous social situation, socially excluded by others, decrease their prosocial behaviors [47], for example, in terms of offering less to others in a Dictator Game [48].

Finally, our results clearly demonstrated that social comparison effects on subsequent cooperative decisions were much more salient to those individuals who independently scored highest on a test of social comparison orientation (SCO). This effect suggests that individual differences in social comparison orientation play a vital role in subsequent decision-making. According to the selective accessibility model [49, 50], social comparison effects may activate two different processing manners: contrast or assimilation. These effects could also extend to the following cooperative decisions depending on the processing of similarity (assimilation process) or dissimilarity (contrast process) of the targets. In the social comparison processes, individuals tend to seek similarities or dissimilarities between self and others. Our results suggest that high SCO individuals might engage processing more broadly and deeply in the similarities of the comparison targets in situations of downward comparison, while focus more on processing dissimilarities of the comparison targets in the upward comparison scenario [51].

In summary, the present study provides novel experimental evidence for the role of social comparison processes on cooperative decision-making. Our design allows us to clearly show that psychological processes based on positive feedback from social comparison promotes increased cooperation in some circumstances and reduced cooperation in others, indicating that social preferences and social emotions play a crucial role in the interpersonal cooperative decision-making. In addition, particular contextual information, such as upward or downward social comparison, can adjust cooperative rates accordingly. Furthermore, individual differences in social comparison orientation mediate the comparison effects in both up- and downward comparison processes. In conclusion, the current results could integrate to the findings of the recent studies that have been conducted to understanding human cooperative behavior [52–54], therefore, not only provide novel evidence for the theories of social comparison, but also provide important implications for our daily life. Future work could usefully extend this behavioral paradigm by exploring the neural mechanisms underlying social comparison processes on cooperation.
